# Personal name in Igbo Culture: A dataset on randomly selected personal names and their statistical analysis

**DOI:** 10.1016/j.dib.2017.08.045

**Published:** 2017-09-01

**Authors:** Hilary I. Okagbue, Abiodun A. Opanuga, Muminu O. Adamu, Paulinus O. Ugwoke, Emmanuela C.M. Obasi, Grace A. Eze

**Affiliations:** a Department of Mathematics, Covenant University, Canaanland, Ota, Nigeria; b Department of Mathematics, University of Lagos, Akoka, Lagos, Nigeia; c Department of Computer Science, University of Nigeria, Nsukka, Nigeria; d Digital Bridge Institute, International Centre for Information & Communications Technology Studies, Abuja, Nigeria; e Computer Science and Informatics Prograamme, Department of Mathematics, Computing & Physical Sciences, Federal University Otuoke, Otuoke, Nigeria; f African Institute for Mathematical Sciences, Cameroon

**Keywords:** Igbo name, Personal name, Statistics, Distribution, Linguistics, Onomastics

## Abstract

This data article contains the statistical analysis of Igbo personal names and a sample of randomly selected of such names. This was presented as the following: 1). A simple random sampling of some Igbo personal names and their respective gender associated with each name. 2). The distribution of the vowels, consonants and letters of alphabets of the personal names. 3). The distribution of name length. 4). The distribution of initial and terminal letters of Igbo personal names. The significance of the data was discussed.

**Specifications Table**

**Table:** 

Subject area	Decision sciences
More specific subject area	Computational Linguistics, pattern analysis in naming
Type of data	Table and MS Excel
How data was acquired	The data was obtained from freely available textbooks, online baby name websites, oral interview, published articles and online discussion forum.
Data format	Raw, partial analyzed
Experimental factors	Simple random sampling of some selected Igbo personal names. The alphabets were presented in their written form (the way they are written in English).
Experimental features	Statistical analysis of the distribution of the following: characters for each name, consonants, vowels, initial letters, terminal letters and total or word length. Comparative ranking of frequency of occurrence.
Data source location	N/A
Data accessibility	All the data are in this data article

**Value of the data**

• The datasets can serve as a reference for Igbo baby names.
• Similar statistical analysis can be applied to other identified names in other languages.
• The dataset can be helpful to the following fields, linguistics, Igbo language studies and lexicology, Anthroponymy, Onomastics, etymology, Igbo name neologism, semantics and morphology of identified Igbo names and so on. See [1] and [2] for research on patterns of writing language texts.
• The data can be used to study the effects of God called “Chi” or “Chukwu” in Igbo personal naming. This can be achieved by studying the occurrence of such names compared with others.
• The data can provide insight on the effect of Christianity and Pentecostalism in Igbo personal naming.
• The reference section can serve as useful resources for researchers in this area.

## 1 Data

The data contained in this article are listed as follows. The dataset of randomly selected Igbo personal names and their respective gender associated with each name, and the distribution of the name length. This data can be assessed as Supplementary data 1. Secondly, the distribution of the vowels, consonants and alphabets of the Igbo personal names was included in this data article.

This data can be assessed as Supplementary data 2. Lastly, this data article contains the distribution of initial and terminal letters of Igbo personal names. In addition, tables showing the statistical analysis of the above listed datasets were also included.

### 1.1 Detailed data description

Personal names can be classified as given name, first name, middle name, forename, Christian name, local name or adopted name. These are opposite of last name, surname, family name or clan name. Igbo is one of the major tribes in Nigeria and the language is spoken by over 25 million and characterized by dialects. The Igbo people are originally from the eastern part of Nigeria but can be found in virtually every country of the world. Similar to any other ethnic groups in Africa, naming in Igbo is premeditated venture that is designed to speak to the future of the newly born child. Igbo people are not careless in naming because of their belief that names are tied to destinies and as such have religious, philosophical, psychological, historical, social and linguistic interpretations. Personal names in Igbo land are characterized by the following: 1). Names are clustered along the lines of dialects, largely because of geographical proximity, migration and historical ties. 2). Sentential names are heavily been replaced with Pentecostal names. 3). The influence of God called “Chi” or “Chukwu” is very strong in Igbo personal names. 4). Superstitious beliefs also influence the naming system. 5). Sociological effects such as procreation and the importance of children over barrenness, wealth, status, riches for example “Nwako”, caste system like “Osu”, “Umeh”, traditional post or monarchial lineage, for example; “Adaeze”, “Ezedinobi”, innuendo or response to mockery, childlessness or taunting for example “Iroahushi”, superiority of their siblings, clan or kingship or kinsmen over others, or their wealth, beauty, riches, sexual or intellectual abilities for example “Akubuilo”, “Ofunneka”. 6). Igbo personal names are gender sensitive because of the patriarchal nature of Igbo people. The males are often named based on issues such as: gods or deities, physical and spiritual objects, intellectual prowess and dexterity in trade or agriculture, natural or mysterious phenomena, sportsmanship and craftsmanship, animals and so on. On the other hand, female names are often associated with good lineage, fruitfulness, beauty and intelligent, moral responsibility, favor, good luck and tidings, joy, happiness, wealth, purity and so on. 7). Maternal lineage or descent can influence the personal names given to Igbo people. 8). Historical or geographical events for example waterfall, market days, birth of prince or princess, disease outbreak, war, famine, draught, great harvest, fruitful period and so on. Numerous investigators have worked on various aspects of personal names and naming in Igbo but the actual distribution and frequency of the alphabets that made up each name have not been reported to the best of the knowledge of the authors.

## 2 Experimental design, materials and methods

This research was as a result of rigorous research gaps observed from the works of numerous authors. Few of which are listed [3], [4], [5], [6], [7], [8], [9], [10], [11], [12], [13], [14], [15], [16], [17], [18], [19], [20], [21], [22], [23], [24], [25], [26], [27], [28], [29], [30], [31], [32], [33], [34], [35], [36], [37], [38], [39], [40], [41], [42], [43], [44], [45], [46], [47], [48].

### 2.1 The random sample of Igbo personal names

The limitations of accessing the target population is compensated with a well-defined sample which must be a true representative of the studied population. See [49], [50], [51], [52], [53], [54], [55], [56], [57], [58], [59], [60], [61], [62], [63], [64], [65], [66], [67] for some selected survey research done to study some observed population attributes. Simple random sampling of some selected Igbo personal names yielded 965 names which are subsets of larger population. The samples were collected in such a way as to reflect the dialectal classification of Igbo people. The data was obtained from freely available textbooks, online baby name websites, oral interview, published articles and online discussion forums.

### 2.2 Distribution of name length of Igbo personal names

Statistical analysis of the personal name (word) length of Igbo people are summarized in Table 1. This was done using simple statistical tools.

**Table 1:** Summary statistics of the distribution of word length of Igbo personal names.

Statistic	Value
Mean	8.34
Median	8
Mode	8
Standard deviation	2.39
Range	17
Minimum	3
Maximum	20
Skewness	0.699
Kurtosis	0.834

**Fig. 1:**
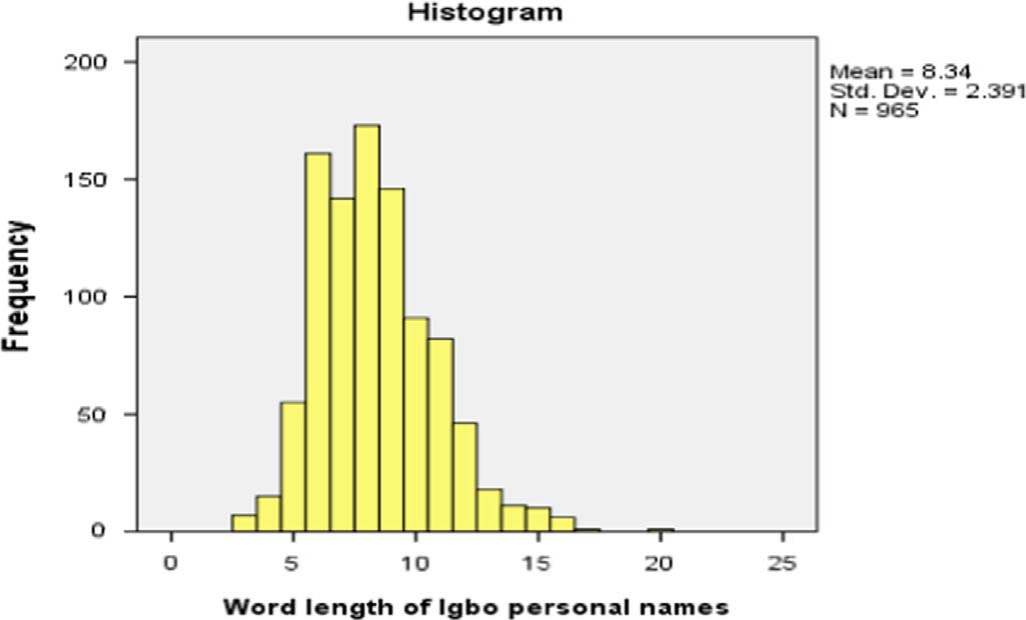
The distribution of Igbo personal name length.

On the average, a randomly selected Igbo personal name will have a word length of eight. The description can be done using histogram as shown in Fig. 1.

It is most likely that the word length will be greater than eight as seen in the histogram (skewness).

### 2.3 Distribution of letters of alphabets and their comparative ranking in Igbo personal names

Igbo language is made up of 36 letters of the alphabets comprising of 8 vowels and 28 consonants. This is shown in Table 2.

**Table 2:** Lower case letters of the alphabets of Igbo language.

a, b, ch, d, e, f, g, gb, gh, gw, h, i, ị, j, k, kp, kw, l, m, n, ṅ, nw, ny, o, ọ, p, r, s, sh, t, u, ụ, v, w, y, z

The research was restricted to 25 letters of the alphabets of the written form of Igbo language (Anglo-Igbo) version which is currently used in child registry, school registration, international passport and national identification and so on. The form is shown in Table 3.

**Table 3:** Lower case letters used for this article (written form in English form).

a, b, c, d, e, f, g, h, i, j, k, l, m, n, ṅ, o, p, r, s, t, u, v, w, y, z

Excel command was used to determine the frequency of letters of alphabets of Igbo personal names. The command is: =SUMPRODUCT(LEN(A2) – LEN(SUBSTITUTE(A2, "letter", ""))). The result was presented along with their corresponding comparative ranks. This is shown in Table 4. The rank is from the most frequent to the least.

**Table 4:** Distribution of letters and their corresponding ranks.

Letter	Frequency	Rank	Letter	Frequency		Rank	Letter	Frequency	Rank
a	914	1	j	58		21	s	87	19
b	216	13	k	514		7	t	59	20
c	457	9	l	155		15	u	808	2
d	231	12	m	406		10	v	0	25
e	766	4	n	565		6	w	324	11
f	56	22	ṅ	4		24	y	100	18
g	129	17	O	572		5	z	137	16
h	494	8	P	28		23			
i	801	3	R	168		14			

Surprisingly none of the 965 names contain the letter "v".

### 2.4 Distribution of double letter consonants in Igbo personal names

Igbo language comprises of nine double letter consonants. These are shown in Table 5.

**Table 5:** Lower case letters of double letter Igbo consonants.

ch, gb, gh, gw, kp, kw, nw, ny, sh

Excel command was used to determine the frequency of double letter consonants of alphabets of Igbo personal names. The command is: =SUMPRODUCT(LEN(A2) – LEN(SUBSTITUTE(A2, "letters", "")))/2. The result was presented along with their corresponding comparative ranks. This is shown in Table 6.

**Table 6:** Distribution of double letters consonants and their corresponding ranks.

Consonant	Frequency	Rank
ch	457	1
gb	27	5
gh	1	8
gw	18	7
kp	20	6
kw	182	2
nw	99	3
ny	75	4
sh	1	8

The high frequency of occurrence of “ch” is a pointer to the influence of God in the naming systems of Igbo people. This is because of the presence of “Chi”, Chukwu, Chuku in almost 50% of Igbo personal names.

### 2.5 Distribution of consonants and vowels in Igbo personal names

**Fig. 2:**
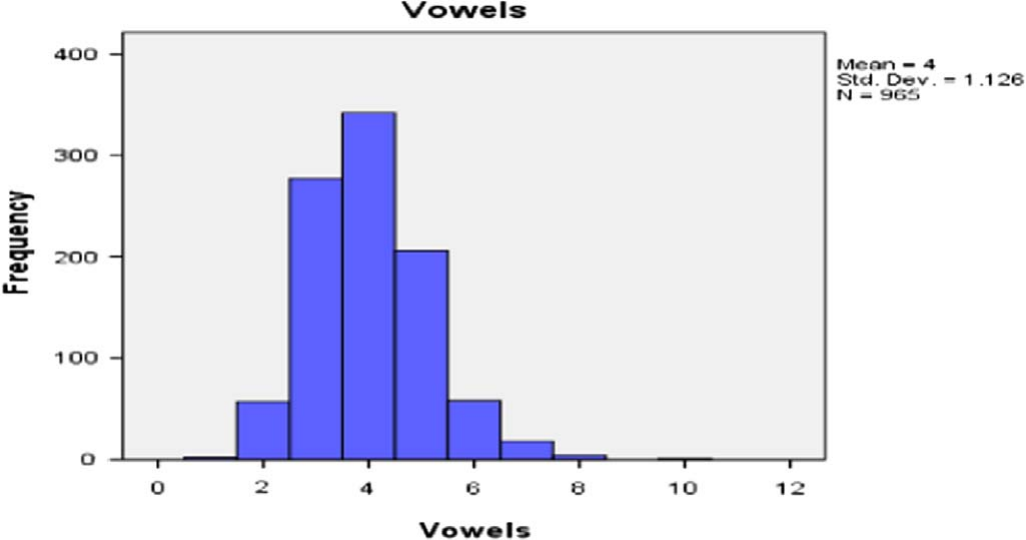
The distribution of vowels in Igbo personal name.

**Fig. 3:**
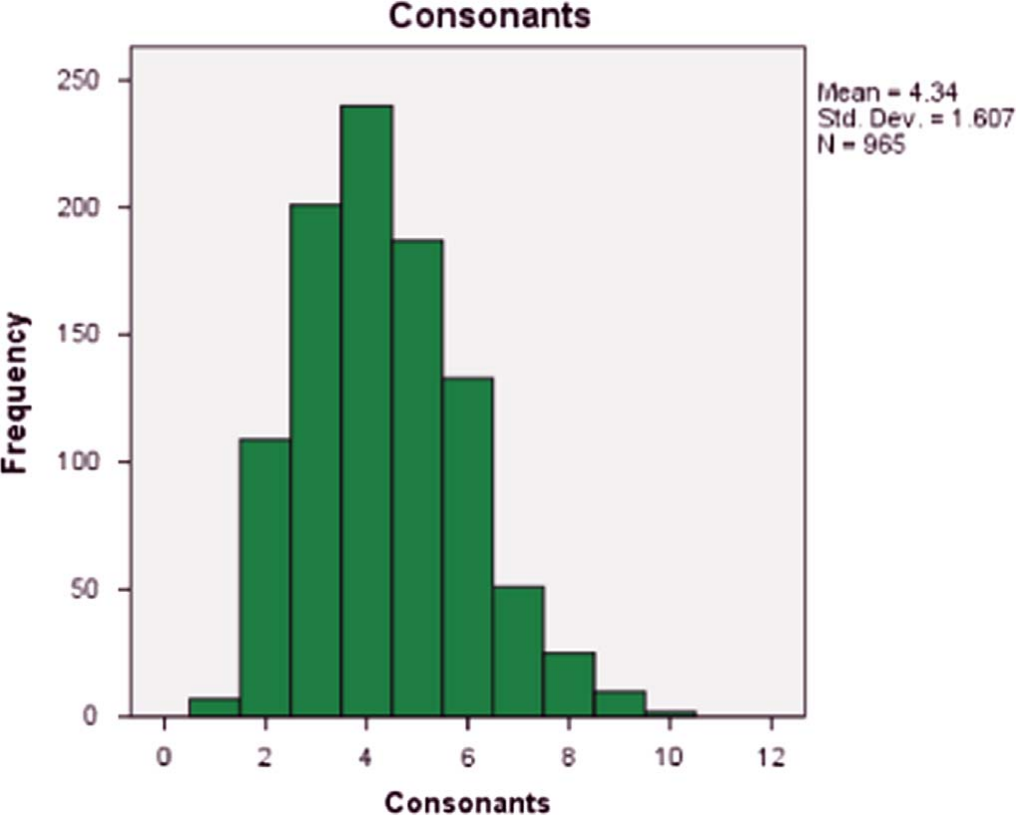
The distribution of consonants in Igbo personal name.

It should observed from Table 4 that the 5 vowels are rank first to fifth and the consonants are ranked after that. However this can be clearly seen in the histogram. The histogram of the distributions of consonants and vowels of 965 randomly selected Igbo personal names are shown in Fig. 2, Fig. 3.

The total number of vowels and consonant and their respective percentages were shown in Table 7. There are a total of 8049 letters. On the average any random selection of Igbo personal name would likely comprised of 48% vowel and 52% consonant.

**Table 7:** Vowels and consonants composition and their percentages.

Vowels	Consonants	Total
3861	4188	8049
48%	52%	100%

### 2.6 Distribution of initial and terminal letters in Igbo personal names and their comparative ranking

Initial and terminal letters constitute a major component of the study of words, nouns, proper nouns and personal names. Excel command was used to determine the frequency of initial and terminal letters of Igbo personal names. The command for the initial letter is: =COUNTIF(A2: A966, “letter*”). The command for the terminal letter is: =COUNTIF(A2: A966, “*letter”). The result was presented along with their corresponding comparative ranks. This is shown in Table 8.

**Table 8:** Distribution of initial and terminal letters and their corresponding ranks.

Letter	Initial	Rank	Terminal	Rank	Letter	Initial	Rank	Terminal	Rank
A	117	3	288	1	n	117	3	1	9
B	2	18.5	0	17.5	ṅ	0	23.5	0	17.5
C	219	1	0	17.5	o	176	2	81	5
D	10	12	0	17.5	p	0	23.5	0	17.5
E	67	7	175	3	r	2	18.5	8	7
F	1	20.5	0	17.5	s	14	10	0	17.5
G	3	16.5	0	17.5	t	4	14	0	17.5
H	0	23.5	3	8	u	80	5	201	2
I	72	6	149	4	v	0	23.5	0	17.5
J	4	14	0	17.5	w	1	20.5	0	17.5
K	26	9	0	17.5	y	3	16.5	0	17.5
L	4	14	0	17.5	z	11	11	0	17.5
M	32	8	58	6					

Areas of similarity and differences and relationship of the initial and terminal letters can be obtained by further analysis and use of statistical methods like correlation and chi-square.
